# Assessment of a prognostic model, PSA metrics and toxicities in metastatic castrate resistant prostate cancer using data from Project Data Sphere (PDS)

**DOI:** 10.1371/journal.pone.0170544

**Published:** 2017-02-02

**Authors:** Bethany Pitcher, Leila Khoja, Robert J. Hamilton, Kald Abdallah, Melania Pintilie, Anthony M. Joshua

**Affiliations:** 1 Department of Biostatistics, Princess Margaret Cancer Centre, University Health Network, Toronto, Ontario, Canada; 2 Department of Medical Oncology, Princess Margaret Cancer Centre, University Health Network, Toronto, Ontario, Canada; 3 Department of Urologic Oncology, University Health Network, Toronto, Ontario, Canada; 4 Project Data Sphere LLC, Cary, North Carolina, United States of America; 5 Department of Medical Oncology, Kinghorn Cancer Centre, St Vincent’s Hospital, Sydney, New South Wales, Australia; Thomas Jefferson University, UNITED STATES

## Abstract

**Background:**

Prognostic models in metastatic castrate resistant prostate cancer (mCRPC) may have clinical utility. Using data from PDS, we aimed to 1) validate a contemporary prognostic model (Templeton et al., 2014) 2) evaluate prognostic impact of concomitant medications and PSA decrease 3) evaluate factors associated with docetaxel toxicity.

**Methods:**

We accessed data on 2,449 mCRPC patients in PDS. The existing model was validated with a continuous risk score, time-dependent receiver operating characteristic (ROC) curves, and corresponding time-dependent Area under the Curve (tAUC). The prognostic effects of concomitant medications and PSA response were assessed by Cox proportional hazards models. One year tAUC was calculated for multivariable prognostic model optimized to our data. Conditional logistic regression models were used to assess associations with grade 3/4 adverse events (G3/4 AE) at baseline and after cycle 1 of treatment.

**Results:**

Despite limitations of the PDS data set, the existing model was validated; one year AUC, was 0.68 (95% CI 95% CI, .66 to .71) to 0.78 (95%CI, .74 to .81) depending on the subset of datasets used. A new model was constructed with an AUC of .74 (.72 to .77). Concomitant medications low molecular weight heparin and warfarin were associated with poorer survival, Metformin and Cox2 inhibitors were associated with better outcome. PSA response was associated with survival, the effect of which was greatest early in follow-up. Age was associated with baseline risk of G3/4 AE. The odds of experiencing G3/4 AE later on in treatment were significantly greater for subjects who experienced a G3/4 AE in their first cycle (OR 3.53, 95% CI 2.53–4.91, p < .0001).

**Conclusion:**

Despite heterogeneous data collection protocols, PDS provides access to large datasets for novel outcomes analysis. In this paper, we demonstrate its utility for validating existing models and novel model generation including the utility of concomitant medications in outcome analyses, as well as the effect of PSA response on survival and toxicity prediction.

## Introduction

It is commonly appreciated that the natural history of prostate cancer varies widely [1–3]. Indeed, there are an increasing array of prognostic tools available in the localized disease setting [4, 5] however, in the metastatic disease state, the utility of prognostic scoring systems has had limited clinical impact in the pre- or post-chemotherapy setting, likely due to the rapid evolution of treatment paradigms (S1 Table) [6–8]. With these advances, the optimal choice of sequencing of treatments is unknown. Furthermore there are no validated predictive biomarkers of response or toxicity with any agent but there are a number of prognostic factors and models that have been derived from both pro- and retrospective studies. For example, previous a meta-analysis of patients treated with docetaxel showed that site of metastatic involvement was the most significant prognostic factor [9]. Other prognostic factors evaluated include lactate dehydrogenase (LDH), hemoglobin, performance status, Gleason score, age, albumin, alkaline phosphatase (ALP), pain, prostate-specific antigen (PSA) doubling time[6, 7, 10] and more recently, the neutrophil-lymphocyte ratio (NLR)[11, 12].

PDS is an non-profit organization [13] that allows registered researchers to access and analyze de-identified patient-level data from comparator arms of phase III clinical trials in cancer. We sought to use PDS data to address outstanding issues in meta-analytic data in prostate cancer care. Our aims were to 1) validate a commonly utilized prognostic model for overall survival to assess prognostic factor importance and applicability 2) determine the effect of concomitant medication on overall survival after accounting for other prognostic variables 3) determine whether a sustained reduction in PSA by a variety of definitions significantly correlates with overall survival and 4) explore if any clinical factors at baseline were predictive of adverse events (AE) on docetaxel treatment.

## Materials and methods

The PDS online database was accessed on December 1^st^ 2014 and data obtained for 2,449 subjects with mCRPC from the control arm of 6 phase III clinical trials (Table 1). For the purposes of analysis; metastatic sites were defined as lymph node (LN) only, bone (with or without LN) and no other metastasis locations, lung (with or without bone/LN), liver (with or without lung and bone/LN), or other. Survival was defined as the time from start of the trial until death. Survival analysis did not include the Prostat_Celgene_2009_90 trial due to missing data.

**Table 1:** List of trials and number of patients (from PDS) used in study analysis.

Clinical Trial	PDS unique dataset ID	Control arm	Number patients
A Phase 3, Randomized, Double-blind, Placebo-Controlled Study of Abiraterone Acetate (CB7630) Plus Prednisone in Patients with Metastatic Castration-Resistant Prostate Cancer Who Have Failed Docetaxel-Based Chemotherapy	Prostat_CougarB_2008_101	Prednisone	387
A Phase 3 Study to Evaluate the Efficacy and Safety of Docetaxel and Prednisone with or without Lenalidomide in Subjects with Castrate-Resistant Prostate Cancer	Prostat_Celgene_2009_90	Docetaxel and prednisone	487
A Phase 3, Randomized, Open-Label Study Evaluating DN-101 in Combination with Docetaxel in Androgen-Independent Prostate Cancer (AIPC) (ASCENT-2)	Prostat_Novacea_2006_89	Docetaxel	427
(VENICE) A Multicenter, Randomized, Double-Blind Study Comparing the Efficacy and Safety of Aflibercept Versus Placebo Administered Every 3 Weeks in Patients Treated with Docetaxel / Prednisone for Metastatic Androgen-Independent Prostate Cancer	Prostat_Sanofi_2007_83	Docetaxel and prednisone	529
A multicenter, randomized, double-blind, phase 3 study of sunitinib plus prednisone versus prednisone in patients with progressive metastatic castration-resistant prostate cancer after failure of a docetaxel-based chemotherapy regimen	Prostat_Pfizer_2008_81	Prednisone	285
A Randomized, Open Label Multi-Center Study of XRP6258 at 25 mg/m^2 in Combination With Prednisone Every 3 Weeks Compared to Mitoxantrone in Combination With Prednisone For The Treatment of Hormone Refractory Metastatic Prostate Cancer	Prostat_Sanofi_2007_79	Mitoxantrone and prednisone	334

The prognostic model proposed by Templeton et al was validated. This model (Templeton et al[11]) was derived retrospectively using non-trial pre-chemotherapy patients and validated with a cohort using selected pre-chemotherapy trial patients from a single center. Templeton et al.’s model was first validated using a data set consisting of two trials that contained all necessary data, CougarB_2008_101, Pfizer_2008_81. A continuous risk score was determined from the parameter estimates of the multivariable model they established. This risk score included the presence of liver metastasis, hemoglobin, alkaline phosphatase (ALP), NLR and lactate dehydrogenase (LDH). We also examined the performance of this model when not all risk factors were available for all datasets, by including all trials in the validation data set and determining the risk score based only on factors that were available across all studies: presence of liver metastasis, hemoglobin and ALP. Here the risk score was determined from the parameter estimates for only these three variables and NLR and LDH were excluded from the risk score calculation.

Performance was assessed with time-dependent receiver operating characteristic (ROC) curves, and the corresponding time-dependent Area Under the Curve (tAUC) at multiple time points (every 3 months) [14]. The 95% Confidence Intervals for the time-dependent AUC were computed using the bootstrap method. Models were compared using the 1 year time point only.

To create a novel model using OS as the endpoint, we first created univariable Cox proportional hazards models, which were fit for each of the 16 clinical covariates, including LDH, ALP, and an indicator variable for more than one organ with metastasis. Backward selection was used to construct a multivariable model. Albumin, LDH, lymphocyte count, NLR, urea and white blood cell count were not collected by some studies producing more than 10% missing values. Thus these variables were not considered in the multivariable model.

Concomitant medications were manually annotated and collated into 38 groups (S2 Table). Non-lipophilic statins and lipophilic statins were analyzed separately as well as a single group. The association of individual concomitant medication with survival was assessed using univariable and multivariable Cox proportional hazards models. The multivariable model was adjusted for all variables in the final multivariable model found in the previous step. The medications taken by fewer than 40 subjects were not considered. To determine if any medications added prognostic value, the time-dependent AUC was calculated for the multivariable model with and without the concomitant medications included.

PSA response was defined in three ways: the percentage PSA decrease from baseline to 12 weeks (PSA12W), and a decrease in PSA (≥30% or ≥50%) that was sustained for at least four weeks, occurring within the first 12 weeks of the start of the study (PSA50 and PSA30). Each PSA response covariate was assessed separately in both univariable and multivariable Cox proportional hazards models. The one-year time-dependent AUC was calculated for each multivariable model. For models including PSA50 or PSA30, subjects with less than 12 weeks of follow-up, whether censored or not, were excluded. This was done in order to avoid potential bias due to shorter follow-up times.

Adverse events of interest on treatment were defined and manually annotated according to the 23 most common adverse events occurring in the TAX327 trial [15] and were considered only for the docetaxel based studies (Table 1). To establish a relationship between the occurrence of a grade 3 or 4 (G3/4) adverse event (AE) and any baseline variables, in patients treated with docetaxel, conditional logistic regression models were fit for 10 variables. Backwards selection was used to construct a multivariable model. Only variables with less than 10% missing values were considered for the multivariable analysis. In a similar fashion, a model was constructed for the occurrence of a G3/4 AE after commencing the second cycle of treatment. Here, the occurrence of a G3/4 AE before the second cycle was also considered as a variable in the multivariable model.

In each of the Cox proportional hazards models, the assumption of proportional hazards was tested for each covariate with the examination of the Schoenfeld residuals. If a covariate was found to violate the proportional hazards assumption, the covariate’s interaction with time was added to the model. To account for differences between the trials, all Cox proportional hazards models and condition logistic regression models were stratified by trial.

## Results

Patient characteristics and baseline prognostic factors were collated including age Gleason score, concomitant medications, sites of metastases, PSA at baseline, albumin, hemoglobin, LDH, ALP, neutrophil count, lymphocyte count and NLR (S3 Table).

### Validation of the prognostic models proposed by Templeton et al

When the entire dataset was used for validation and only a subset of the variables were available to calculate the risk score, the one-year tAUC was .68 (95% CI, .66 to .71) (S1 Fig). In this case the risk score was calculated utilizing only the available covariates.

### Multivariable model

The final model (incorporating all available parameters defined by Templeton et al., in combination with the available data fields across all the studies) included, hemoglobin, ALP, neutrophil count and organ involvement, i.e. the number of organs with metastases (>1 vs. 1 or less) (Table 2). A model that included metastasis location, instead of the number of metastases, had similar performance, with one-year tAUC equal to .75 (.73 to .78), as opposed to .74 (.72 to .77) for the previously chosen model. The model including the number of metastases was preferred as it was the more parsimonious of the two models. Metastasis location and number of metastases cannot be included in the same model as they are collinear; the number of metastases sites is determined as the sum of the metastasis locations.

**Table 2:** Univariable and Multivariable Models predicting Overall Survival (n = 1935).

		*Univariable analysis*		*Multivariable Analysis*	
	missing	HR (95%CI)	p-value	HR (95%CI)	p-value
*ALBUMIN*, *g/dL*	791 (40.9%)	0.4 (0.4–0.5)	< .0001		
*log(ALP*,*U/L)*	38 (2%)	1.6 (1.5–1.7)	< .0001	1.52 (1.42–1.63)	< .0001
*BSA*	41 (2.1%)	0.6 (0.5–0.9)	0.0071		
*log(Creatinine*, *mg/dL)*	56 (2.9%)	0.9 (0.7–1.2)	0.47		
*HEMOGLOBIN*, *g/dL*	78 (4%)	0.8 (0.7–0.8)	< .0001	0.81 (0.77–0.85)	< .0001
*log(LDH*, *U/L)*	914 (47.2%)	3.2 (2.8–3.7)	< .0001		
*log(LYMPHOCYTES*, 10*3/UL)	997 (51.5%)	0.5 (0.4–0.6)	< .0001		
**Metastasis**					
*BONE vs LN*	14 (0.7%)	1.2 (0.9–1.5)	0.28		
*LUNG vs LN*	14 (0.7%)	1.2 (0.9–1.7)	0.18		
*LIVER vs LN*	14 (0.7%)	2.3 (1.7–3.1)	< .0001		
*OTHER vs LN*	14 (0.7%)	1.5 (1.1–2)	0.013		
*log(Neutrophil*, 10*3/UL)	79 (4.1%)	1.6 (1.4–1.8)	< .0001	1.51 (1.3–1.75)	< .0001
*log(NLR)*	997 (51.5%)	1.7 (1.5–1.9)	< .0001		
*> 1 organ*	14 (0.7%)	1.4 (1.2–1.6)	< .0001	1.46 (1.28–1.66)	< .0001
*log(Platelets*,10*3/UL)	118 (6.1%)	1.3 (1.1–1.6)	0.0046		
*log(PLR)*	1036 (53.5%)	1.8 (1.5–2.1)	< .0001		
**RACE**					
*Asian vs*. *White*	458 (23.7%)	1 (0.8–1.3)	0.95		
*Black vs White*	458 (23.7%)	0.8 (0.6–1.2)	0.32		
*Other vs*. *White*	458 (23.7%)	0.9 (0.7–1.3)	0.74		
*log(Urea*, *mg/dL)*	1323 (68.4%)	1.2 (0.8–1.6)	0.38		
*log(WBC)*	379 (19.6%)	1.3 (1.1–1.6)	0.015		

### Concurrent medication

Of the 38 medication types, only 22 were common enough to be considered. In the unadjusted, univariable analysis erythropoietin, fish oil, prazoles, non-lipophilic statins and warfarin were prognostic (Table 3). After adjusting for the factors determined to be significant from the combined model; ALP, hemoglobin, neutrophil count, and organ involvement, the use of LMWH, and warfarin were associated with poorer survival whilst Metformin and Cox2 inhibitors were associated with better outcome (Table 3). There was no evidence that the effect of Metformin differed with increasing BSA or weight (data not shown). However, adding medication use to the multivariable model established above did not improve the predictive ability. With the addition of LMWH, the 1-year tAUC was .74(95% CI, .72 to .77), adding warfarin the tAUC was .74(95% CI, .72 to .77), adding Metformin resulted in a tAUC of .73(95% CI, .71 to .76), and adding Cox2 inhibitors resulted in a one year tAUC of .74(95% CI, .72 to .77). The addition of all four medications to the multivariable model resulted in a tAUC of .74 (95% CI, .72 to .77) at one year (S2 Fig), identical to the model without the medications.

**Table 3:** Unadjusted Prognostic effect (n = 1935) and adjusted prognostic effect (n = 1828) of concomitant medications on overall survival (Adjusted for the effect of hemoglobin, ALP, neutrophil count and organ involvement).

	Number of subjects on medication (%)	Unadjusted HR (95% CI)	Unadjusted p-value	Adjusted HR (95%CI)	Adjusted p-value
ASPIRIN	463 (23.6%)	0.87 (0.76–1.01)	0.064	0.97 (0.84–1.12)	0.69
BETABLOCKER	393 (20%)	1 (0.86–1.16)	0.98	1.03 (0.89–1.21)	0.66
CALCIUM	351 (17.9%)	1.02 (0.87–1.19)	0.79	1 (0.85–1.17)	0.98
COX2INH	113 (5.8%)	0.82 (0.63–1.07)	0.14	0.69 (0.52–0.91)	**0.0077**
DIGOXIN	43 (2.2%)	1.04 (0.7–1.56)	0.84	0.95 (0.62–1.44)	0.81
EPO	194 (9.9%)	1.45 (1.19–1.78)	**0.00024**	1.09 (0.88–1.35)	0.41
ESTROGEN	227 (11.6%)	0.91 (0.76–1.08)	0.26	0.91 (0.76–1.09)	0.31
FISH_OIL	71 (3.6%)	0.67 (0.47–0.95)	**0.025**	0.73 (0.51–1.06)	0.098
GCSF	67 (3.4%)	0.88 (0.65–1.2)	0.43	0.9 (0.66–1.23)	0.51
INSULIN	120 (6.1%)	1.06 (0.83–1.35)	0.65	0.98 (0.75–1.27)	0.87
KETOCONAZOLE	159 (8.1%)	0.9 (0.73–1.1)	0.3	0.86 (0.7–1.06)	0.16
LIPOPHILIC_STATIN	382 (19.5%)	0.95 (0.82–1.11)	0.54	1.05 (0.9–1.22)	0.57
LMWH	170 (8.7%)	1.21 (1–1.47)	0.054	1.28 (1.05–1.57)	**0.016**
METFORMIN	174 (8.9%)	0.84 (0.68–1.05)	0.12	0.79 (0.63–0.99)	**0.043**
NITRATE	72 (3.7%)	0.98 (0.71–1.34)	0.88	0.99 (0.71–1.37)	0.94
NON_LIPOPHILIC_STATIN	75 (3.8%)	0.67 (0.49–0.93)	**0.017**	0.8 (0.57–1.13)	0.21
NSAID	712 (36.3%)	1.11 (0.98–1.25)	0.1	0.96 (0.85–1.09)	0.51
PRAZOLE	786 (40.1%)	1.17 (1.04–1.31)	**0.011**	1.12 (0.99–1.27)	0.061
SELENIUM	46 (2.3%)	0.82 (0.53–1.26)	0.36	1.01 (0.65–1.58)	0.95
STATIN	449 (22.9%)	0.88 (0.76–1.02)	0.08	1 (0.86–1.15)	0.95
SULFONLUREA	135 (6.9%)	1.1 (0.89–1.37)	0.38	0.96 (0.77–1.21)	0.74
VIT_C	74 (3.8%)	0.84 (0.61–1.15)	0.27	0.9 (0.64–1.25)	0.53
WARFARIN	190 (9.7%)	1.22 (1–1.49)	**0.048**	1.26 (1.02–1.54)	**0.03**

### PSA response

PSA12W was associated with survival (Table 4). Both before and after adjusting for adjusting for ALP, hemoglobin, neutrophil count and more than one disease site, patients who experienced greater reductions in PSA had a significantly lower risk of death. PSA50 and PSA30 were found to violate the proportional hazards assumption. In both the univariable and multivariable analysis, patients who had a sustained decrease in PSA of 30% or 50%, within 12 weeks of the start of trial, had a lower risk of death (Table 4). However, the effect of a PSA decrease on survival was greatest early in follow-up and diminished with increasing time (Table 4). The one-year tAUCs did not differ significantly between the multivariable models with PSA12W, PSA50 and PSA30, indicating that no variable outperformed the others in terms of predictive accuracy.

**Table 4:** Unadjusted Prognostic effect and adjusted prognostic effect of PSA on overall survival.

variable	n (adjusted model)	Unadjusted HR (95% CI)	Unadjusted p-value	Adjusted HR (95%CI)	Adjusted p-value	tAUC (1 year)
log(1—*PSA*_12weeks_)	1220	1.5 (1.38–1.63)	< .0001	1.49 (1.37–1.62)	< .0001	0.81 (0.78–0.84)
PSA50 (No Response vs. Response)	1527	4.43 (2.92–6.71)	< .0001	3.77 (2.48–5.73)	< .0001	0.77 (0.74–0.8)
PSA50 with time		1 (1–1)	< .0001	1 (1–1)	0.0004	
PSA30 (No Response vs. Response)	1516	5.52 (3.69–8.27)	< .0001	5.02 (3.33–7.56)	< .0001	0.78 (0.76–0.81)
PSA30 with time		1 (1–1)	< .0001	1 (1–1)	< .0001	

### Evaluation of the association of docetaxel treatment with toxicity

Of the 1442 patients treated with docetaxel, 1259 (87%) experienced at least one AE and 417 (29%) experienced at least one G3/4 AE (Table 5). The risk for a G3/4 AE (Table 6) was found to be associated only with age in a multivariable analysis. Of the 1366 patients who received more than one cycle of medication, 184 (13%) experienced a G3/4 adverse event before the start of the second cycle. The odds of experiencing a severe AE later on in treatment were over 3 times greater for subjects who experienced a G3/4 AE in their first cycle of treatment (OR 3.53, 95% CI 2.53–4.91, p < .0001, Table 6).

**Table 5:** Distribution of maximum toxicity grade experienced by patient across AE categories.

	None	Grade 1	Grade 2	Grade 3	Grade 4	Grade 5
*Alopecia*	821 (57%)	433 (30%)	186 (13%)	3 (0%)	0 (0%)	0 (0%)
*Anemia*	1213 (84%)	107 (7%)	81 (6%)	34 (2%)	8 (1%)	0 (0%)
*Anorexia*	1228 (85%)	136 (9%)	71 (5%)	8 (1%)	0 (0%)	0 (0%)
*Change in Taste*	1126 (78%)	267 (19%)	47 (3%)	3 (0%)	0 (0%)	0 (0%)
*LVEF Decrease*	1441 (100%)	0 (0%)	2 (0%)	0 (0%)	0 (0%)	0 (0%)
*Diarrhea*	1045 (72%)	252 (17%)	108 (7%)	33 (2%)	5 (0%)	0 (0%)
*Dyspnea*	1224 (85%)	137 (9%)	60 (4%)	22 (2%)	0 (0%)	0 (0%)
*Epistaxis*	1359 (94%)	77 (5%)	6 (0%)	1 (0%)	0 (0%)	0 (0%)
*Fatigue*	778 (54%)	328 (23%)	261 (18%)	72 (5%)	4 (0%)	0 (0%)
*Febrile Neutropenia*	1353 (94%)	1 (0%)	2 (0%)	58 (4%)	28 (2%)	1 (0%)
*Myalgia*	1348 (93%)	59 (4%)	33 (2%)	3 (0%)	0 (0%)	0 (0%)
*Nausea*, *Vomiting*, *or both*	1109 (77%)	235 (16%)	86 (6%)	11 (1%)	2 (0%)	0 (0%)
*Nail Changes*	1113 (77%)	229 (16%)	93 (6%)	8 (1%)	0 (0%)	0 (0%)
*Neutropenia*	1186 (82%)	24 (2%)	33 (2%)	85 (6%)	115 (8%)	0 (0%)
*Peripheral Edema*	1046 (72%)	272 (19%)	115 (8%)	10 (1%)	0 (0%)	0 (0%)
*Sensory Neuropathy*	957 (66%)	327 (23%)	120 (8%)	39 (3%)	0 (0%)	0 (0%)
*Stomatitis*	1234 (86%)	137 (9%)	60 (4%)	12 (1%)	0 (0%)	0 (0%)
*Tearing*	1295 (90%)	127 (9%)	21 (1%)	0 (0%)	0 (0%)	0 (0%)
*Thrombocytopenia*	1410 (98%)	25 (2%)	2 (0%)	5 (0%)	1 (0%)	0 (0%)

**Table 6:** Multivariable Conditional Logistic Regression for occurrence of a severe AE (Grade ≥3) (n = 1442) and for occurrence of a severe AE (Grade ≥3) after start of Cycle 2 (n = 1366).

		Occurrence of any severe AE (Grade ≥3)				Occurrence of a severe AE (Grade ≥3) after start of Cycle 2			
		*Univariable analysis*		*Multivariable Analysis*		*Univariable analysis*		*Multivariable Analysis*	
* *	*Missing (%)*	*OR (95% CI)*	*p-value*	*OR (95% CI)*	*p-value*	*OR (95% CI)*	*p-value*	*OR (95% CI)*	*p-value*
*Age*, *years*	0 (0%)	1.05 (1.03–1.07)	< .0001	1.05 (1.03–1.07)	< .0001	1.05 (1.03–1.06)	< .0001	1.04 (1.02–1.06)	< .0001
*ALBUMIN*, *g/dL*	454 (31.5%)	0.57 (0.39–0.83)	0.003			0.7 (0.47–1.04)	0.08		
*log(ALP*,*U/L)*	24 (1.7%)	0.89 (0.77–1.02)	0.1			0.89 (0.76–1.04)	0.1		
*BSA*	10 (0.7%)	0.92 (0.5–1.69)	0.8			1.01 (0.53–1.96)	1		
*log(Creatinine*, *mg/dL)*	24 (1.7%)	0.94 (0.59–1.52)	0.8			1.26 (0.76–2.1)	0.4		
*AE (Grade ≥ 3) occurring before Cycle 2*						3.83 (2.76–5.31)	< .0001	3.53 (2.53–4.91)	< .0001
*log(LDH*, *U/L)*	561 (38.9%)	1.04 (0.75–1.44)	0.8			1.04 (0.72–1.49)	0.8		
*log(LYMPHOCYTES*, 10*3/UL)	987 (68.4%)	0.68 (0.44–1.05)	0.08			0.92 (0.58–1.48)	0.7		
*Pain or Fracture occurring before start of treatment*	14 (1%)	1.11 (0.8–1.55)	0.5			1.25 (0.88–1.77)	0.2		
*log(Urea*, *mg/dL)*	553 (38.3%)	1.77 (1.06–2.98)	0.03			2.21 (1.26–3.85)	0.005		

## Discussion

The trials accessed from PDS included 4 pre-chemotherapy and 2 post-chemotherapy trials. The pooled patient data was representative of a good prognostic population; the majority had bone and or nodal disease and had good performance status (ECOG 0–1) with normal range biochemical parameters.

We validated a recent prognostic model using PDS data, which provides confirmation of its utility. The model had reasonably good performance even when not all covariates were available and that the patient characteristics differed from those of the original studies. Templeton et al’s original prognostic score was determined by assigning one risk point for each significant variable in the multivariable regression model they established. Instead of using this approach, we calculated the prognostic score from the parameter estimates of their model as we believed it would provide better discriminatory ability. However, in an unreported analysis, we found that Templeton’s simpler approach yielded very similar results. This further emphasizes the usefulness of this model, as it is applicable in varied populations and performs well with only a subset of the required information. Interestingly, this model incorporates NLR to be an additional independent prognostic marker. The importance of NLR as a reflection of the host-tumor interaction has been increasingly recognized across a number of malignancies lately [16–18], however in our larger dataset we were only able to robustly analyze neutrophil count (due to missing data), which was nevertheless significant. This may suggest that it is the detrimental effects of an inflammatory response that drives the poor prognostic effect of either the neutrophil count or the NLR. Other variables in our combined model are well appreciated such as hemoglobin and ALP, and are likely a reflection of the bulk of the disease, as is the number of metastases. Previously, published post-chemotherapy models have used response to previous treatments such as docetaxel as a prognostic factor [19]. Whilst the value of the response to previous treatments has been recognized in a number of prognostic models (e.g. duration of hormone use, duration of docetaxel administration) we sought to develop a model agnostic to previous treatments given the heterogeneity in the dataset and in order to increase the utility of the model. We defined survival as time from trial entry to death meaning that OS was dependent on the length of follow up on trial. There was no data uniformly available in PDS on date of diagnosis with mCRPC to enable use of a different time point.

The findings on concomitant medication (e.g. Metformin and cox-2 inhibitors were associated with better prognosis) are novel and suggest a possible therapeutic role for certain concomitant medications although this will require dedicated studies. The Stampede study (clinicaltrials.gov NCT00268476) in early de novo metastatic castrate sensitive prostate cancer did not find any therapeutic benefit from celecoxib alone although the different patient population to that studied here may account for our discrepant findings [20]. Nevertheless Cox-2 inhibitors have been associated with the broad range of anti-cancer properties such as apoptosis induction [21] and down-regulation of kinetochore/centromere proteins [22]. Metformin has been tested in a small phase 2 study in chemotherapy naïve castration-resistant prostate cancer, where 2/36 (~5%) of patients had a 50% PSA response [23]. It has also been associated with several anti-cancer mechanisms of action in prostate cancer including inhibition of complex 1[24], lipogenesis [25], decreases of cyclin D1[26], down-regulation of the insulin-like growth factor 1 receptor [27] and AR downregulation[28]. The role of anti-coagulants such as warfarin and LMWH in prostate cancer remain controversial in the literature. Our study found a detrimental effect of warfarin however other retrospective reviews have found inconclusive or no effects of warfarin [29–31]. However, a recent single institution retrospective series of LMWH treated men (for deep venous thrombosis only) with docetaxel (17/247) suggested benefit on multi-variate analysis (HR, 0.48; *P* = .035) [19]. We anticipate that the population in our study had cardiac or vascular comorbidities that significantly compromised their survival.

PSA metrics as parameters of response or surrogates for survival in prostate cancer have remained controversial depending on the trial context and power of the analysis [32]. We looked at the value of a variety of PSA metrics found that variables with the most accurate association with survival was with an estimate of the extent of PSA fall (PSA12W0) rather than the presence or absence of a certain percentage of PSA fall. However, despite these correlations, the AUC for survival at 1 year was not significantly different between models, suggesting that individual level discrimination between the PSA parameters was not sufficient, given the impact of other disease variables.

Appropriate cessation of chemotherapy for toxicity or lack of clinical benefit is used as a benchmark of quality of care in oncology [33]. Whilst predictive scores of toxicity and mortality have been proposed particularly in the elderly population, none have been validated in the clinic [34]. Selection of patients for treatment and optimal management based on these models remains problematic. Our modeling of toxicity confirms clinical acumen in that (i) elderly men are more likely to experience toxicities from chemotherapy, although previous clinical series suggest that docetaxel is very tolerable in the elderly and (ii) that patients that have experienced one adverse event are likely to experience further events.

The analysis herein has inherent limitations such as the quality and completeness of data across trial cohorts and heterogeneity of trial populations. Our findings suggest that PDS can provide a novel platform for large-scale analysis to address effect sizes and questions that are not feasible in single trials.

## Supporting information

S1 Fig Time Dependency of Model. Time-dependent area under the curve (AUC) for the prognostic model proposed by Templeton et al al evaluated on Cougar and Pfizer datasets (Validation 1) and all available datasets (Validation 2).
(DOCX)

S2 Fig Time Dependency of Final Model. Time-dependent area under the curve (AUC) for the final multivariable model, with and without the inclusion of LMWH, metformin, Cox 2 Inhibitors, and warfarin. The final multivariable model includes alkaline phosphatase, hemoglobin, neutrophil count and more than one disease site.
(DOCX)

S1 Table Prognostic Factors. A list of prognostic factors derived from previous studies and prognostic scores proposed thereof in CRPC.
(DOC)

S2 Table Concurrent Medications. Concurrent medication in the 6 trials from Project Data Sphere (shaded indicates medications that were used in analysis).
(DOC)

S3 Table Baseline Clinical Factors. Baseline clinical factors in the 6 trials from Project Data Sphere.
(DOC)
