# Supplementary figures and images for: Assessment of a prognostic model, PSA metrics and toxicities in metastatic castrate resistant prostate cancer using data from Project Data Sphere (PDS)
Source: PLoS One. 2017 Feb 2;12(2):e0170544. doi: 10.1371/journal.pone.0170544 (PMC5289419; doi:10.1371/journal.pone.0170544)

**S1 Figure.**


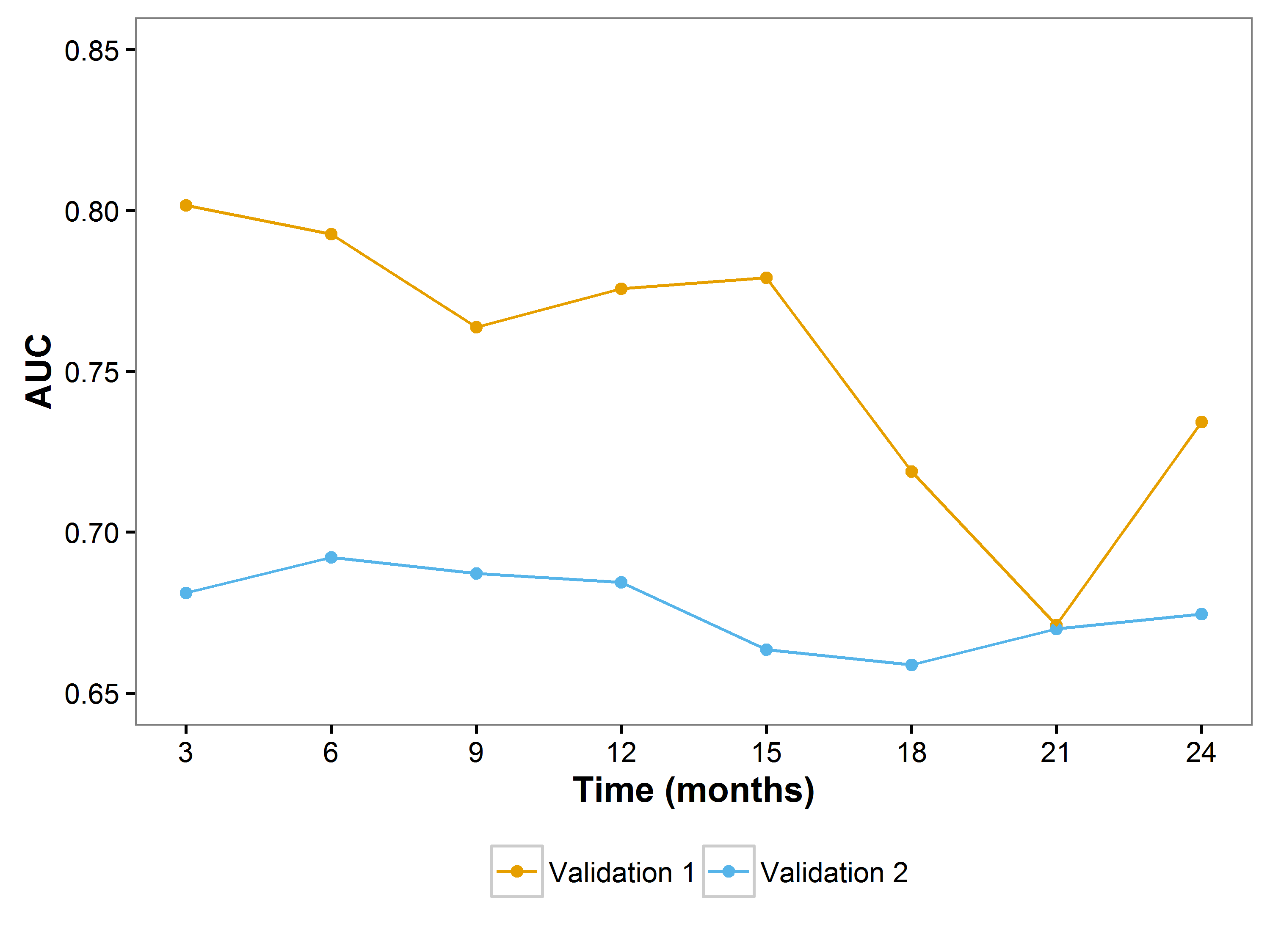

Supplement: S1 Fig — Time-dependent area under the curve (AUC) for the prognostic model proposed by Templeton et al al evaluated on Cougar and Pfizer datasets (Validation 1) and all available datasets (Validation 2). (DOCX) [file pone.0170544.s001.docx]

**S2 Figure.**


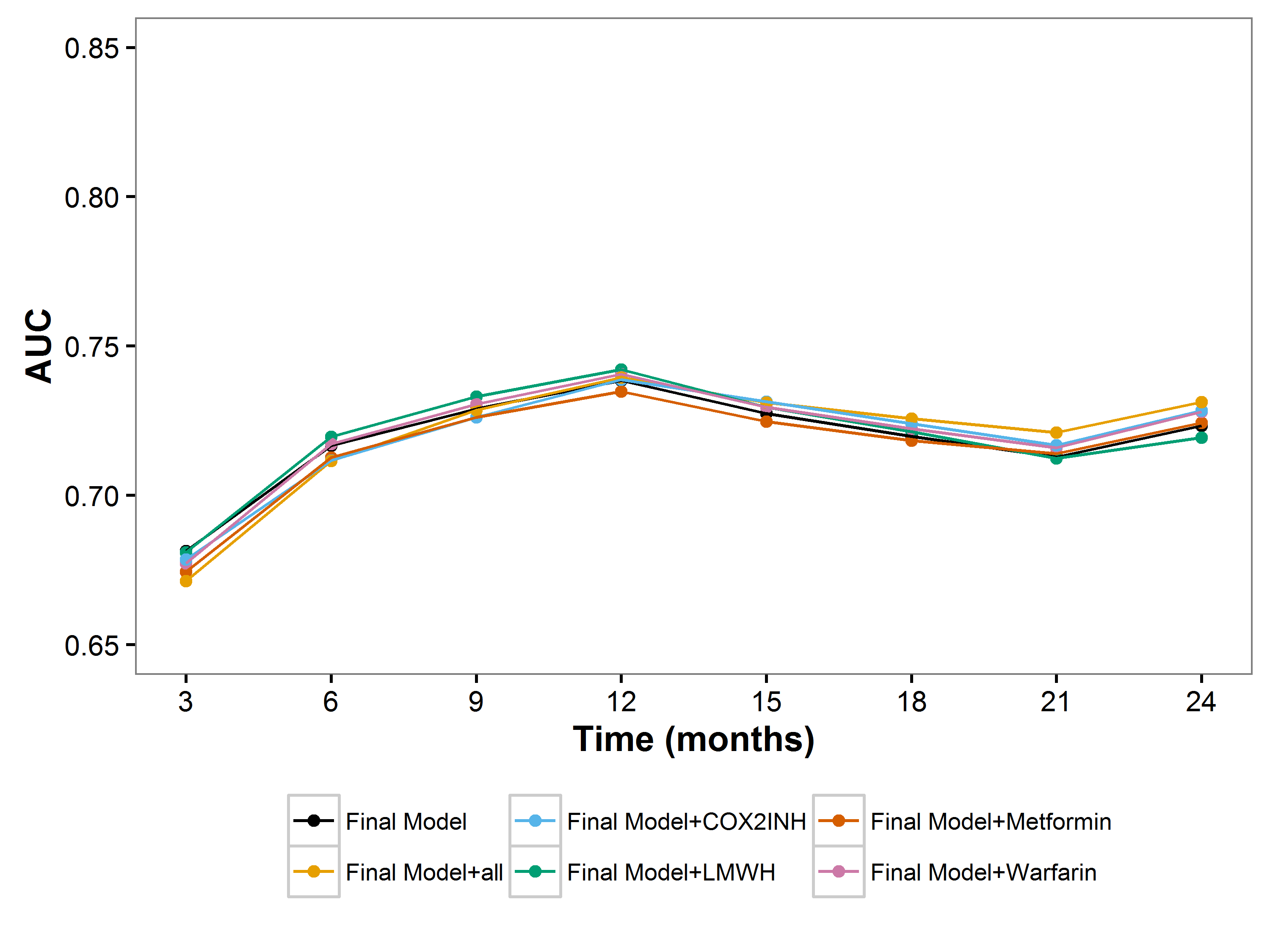

Supplement: S2 Fig — Time-dependent area under the curve (AUC) for the final multivariable model, with and without the inclusion of LMWH, metformin, Cox 2 Inhibitors, and warfarin. The final multivariable model includes alkaline phosphatase, hemoglobin, neutrophil count and more than one disease site. (DOCX) [file pone.0170544.s002.docx]
