# Supplementary material for: Assessment of a prognostic model, PSA metrics and toxicities in metastatic castrate resistant prostate cancer using data from Project Data Sphere (PDS)
Source: PLoS One. 2017 Feb 2;12(2):e0170544. doi: 10.1371/journal.pone.0170544 (PMC5289419; doi:10.1371/journal.pone.0170544)
Supplement: S1 Table — A list of prognostic factors derived from previous studies and prognostic scores proposed thereof in CRPC. (DOC) [file pone.0170544.s003.doc]

S1 Table.

|  | **Smaletz (2002)**  **Nomogram**  **N=409** | **Armstrong (2007)**  **N=1006** | **Halabi (2014)**  **Nomogram N=1050** | **Templeton(2014)**  **Score n=572** | **Halabi (2013)**  **N=1243**  **Nomogram** | **Sonpavde(2014)**  **N=784** |
| --- | --- | --- | --- | --- | --- | --- |
| Clinical setting | Pre-chemotherapy | Pre-chemotherapy | Pre-chemotherapy | Pre-chemotherapy | Post docetaxel chemotherapy | Post docetaxel chemotherapy |
| Non-visceral disease | - | - | Lymph node, bone | - | - | - |
| Visceral involvement | - | Liver metastases | Liver, Lung, adrenal | Liver | Yes/no | ≥1 organ involvement |
| Concurrent medication | - | - | Opioid analgesic use | - | - | - |
| Age | 40-85 | - | - | - | - | - |
| LDH | 1955-116 | - | >ULN | >1.2xULN | - | Log LDH |
| Karnofsky/ECOG performance status | 50-90 | <70 vs ≥80 | 0-2 | - | 0-2 | - |
| Hemoglobin (g/dl) | 6-17 | 18-8 | 17-7 | <120 | 19-7 | Hemoglobin |
| Alkaline Phosphatase (U/L) | 3079-19 | 0-5000 | 33-4915 | >2.0x ULN | 1-8103 | Log ALP |
| Albumin (g/dl) | 2.6-5.2 | - | 6-1 | - | - | - |
| PSA (ng/ml) | 0-8450 | 0-5000 | 0-8103.1 | - | 1-8103 | Log PSA |
| Neutrophil/lymphocyte ratio (NLR) | - | - | - | >3.0 | - | Log NLR |
| Previous docetaxel | - | - | - | - | - | Log number of docetaxel cycles |
| Progression on docetaxel | - | - | - | - | <6 months (yes/no) | yes/no |
| Other factors | - | Number metastatic sites (>2 vs ≤2), pain at baseline (Yes/No), chemotherapy type, measureable disease vs bone only/ tumor grade/ baseline PSA doubling time | - | - | Measurable disease (yes/no), pain at baseline (yes/no), duration on hormone therapy | - |
| Prognostic groupings | Median 1 year, 2 year survival estimates | Median overall survival and 1 year, 2 year and 5 year survival estimates | Low, intermediate, high risk | Very low, low, intermediate, high | Low, intermediate, high risk | Low, medium, high |
