# Supplementary material for: Assessment of a prognostic model, PSA metrics and toxicities in metastatic castrate resistant prostate cancer using data from Project Data Sphere (PDS)
Source: PLoS One. 2017 Feb 2;12(2):e0170544. doi: 10.1371/journal.pone.0170544 (PMC5289419; doi:10.1371/journal.pone.0170544)
Supplement: S2 Table — Concurrent medication in the 6 trials from Project Data Sphere (shaded indicates medications that were used in analysis). (DOC) [file pone.0170544.s004.doc]

S2 Table.

|  | *Celgene_2009_90*  ***(n=487)*** | *CougarB_2008_101*  ***(n=387)*** | *Novacea_2006_89*  ***(n=427)*** | *Pfizer_2008_81*  ***(n=285)*** | *Sanofi_2007_79*  ***(n=334)*** | *Sanofi_2007_83*  ***(n=529)*** | *All*  ***(n=2449)*** |
| --- | --- | --- | --- | --- | --- | --- | --- |
| **ACETYLCYSTEINE** | 1 (0.2%) | 5 (1.3%) | 4 (0.9%) | 3 (1.1%) | 5 (1.5%) | 18 (3.4%) | 36 (1.5%) |
| **AMLODIPINE** | 0 (0%) | 0 (0%) | 0 (0%) | 1 (0.4%) | 0 (0%) | 0 (0%) | 1 (0%) |
| **ASPIRIN** | 76 (15.6%) | 102 (26.4%) | 115 (26.9%) | 55 (19.3%) | 73 (21.9%) | 118 (22.3%) | 539 (22%) |
| **BETABLOCKER** | 95 (19.5%) | 84 (21.7%) | 106 (24.8%) | 44 (15.4%) | 61 (18.3%) | 98 (18.5%) | 488 (19.9%) |
| **CALCIUM** | 71 (14.6%) | 106 (27.4%) | 73 (17.1%) | 51 (17.9%) | 55 (16.5%) | 66 (12.5%) | 422 (17.2%) |
| **CLODRONATE** | 7 (1.4%) | 3 (0.8%) | 2 (0.5%) | 8 (2.8%) | 4 (1.2%) | 17 (3.2%) | 41 (1.7%) |
| **COLCHICINE** | 0 (0%) | 2 (0.5%) | 1 (0.2%) | 1 (0.4%) | 0 (0%) | 2 (0.4%) | 6 (0.2%) |
| **COX2INH** | 11 (2.3%) | 26 (6.7%) | 20 (4.7%) | 24 (8.4%) | 17 (5.1%) | 26 (4.9%) | 124 (5.1%) |
| **DIGOXIN** | 13 (2.7%) | 7 (1.8%) | 16 (3.7%) | 2 (0.7%) | 5 (1.5%) | 13 (2.5%) | 56 (2.3%) |
| **DIPYRIMADOLE** | 3 (0.6%) | 0 (0%) | 0 (0%) | 0 (0%) | 1 (0.3%) | 5 (0.9%) | 9 (0.4%) |
| **DOXAZOSIN** | 5 (1%) | 4 (1%) | 3 (0.7%) | 12 (4.2%) | 2 (0.6%) | 7 (1.3%) | 33 (1.3%) |
| **EPO** | 16 (3.3%) | 16 (4.1%) | 100 (23.4%) | 13 (4.6%) | 44 (13.2%) | 21 (4%) | 210 (8.6%) |
| **ESTROGEN** | 1 (0.2%) | 52 (13.4%) | 1 (0.2%) | 30 (10.5%) | 47 (14.1%) | 97 (18.3%) | 228 (9.3%) |
| **EZETIMIBE** | 8 (1.6%) | 12 (3.1%) | 2 (0.5%) | 4 (1.4%) | 6 (1.8%) | 12 (2.3%) | 44 (1.8%) |
| **FINASTERIDE** | 4 (0.8%) | 6 (1.6%) | 10 (2.3%) | 2 (0.7%) | 2 (0.6%) | 6 (1.1%) | 30 (1.2%) |
| **FISH_OIL** | 15 (3.1%) | 25 (6.5%) | 15 (3.5%) | 4 (1.4%) | 8 (2.4%) | 19 (3.6%) | 86 (3.5%) |
| **GCSF** | 15 (3.1%) | 1 (0.3%) | 6 (1.4%) | 1 (0.4%) | 26 (7.8%) | 33 (6.2%) | 82 (3.3%) |
| **GEMFIBROZIL** | 3 (0.6%) | 1 (0.3%) | 3 (0.7%) | 0 (0%) | 2 (0.6%) | 2 (0.4%) | 11 (0.4%) |
| **GLITAZONE** | 3 (0.6%) | 9 (2.3%) | 15 (3.5%) | 1 (0.4%) | 7 (2.1%) | 7 (1.3%) | 42 (1.7%) |
| **INSULIN** | 18 (3.7%) | 18 (4.7%) | 39 (9.1%) | 13 (4.6%) | 19 (5.7%) | 31 (5.9%) | 138 (5.6%) |
| **ITRACONAZOLE** | 0 (0%) | 0 (0%) | 0 (0%) | 0 (0%) | 2 (0.6%) | 1 (0.2%) | 3 (0.1%) |
| **KETOCONAZOLE** | 3 (0.6%) | 11 (2.8%) | 8 (1.9%) | 17 (6%) | 59 (17.7%) | 64 (12.1%) | 162 (6.6%) |
| **LIPOPHILIC_STATIN** | 68 (14%) | 88 (22.7%) | 100 (23.4%) | 44 (15.4%) | 54 (16.2%) | 96 (18.1%) | 450 (18.4%) |
| **LMWH** | 10 (2.1%) | 47 (12.1%) | 21 (4.9%) | 8 (2.8%) | 36 (10.8%) | 58 (11%) | 180 (7.3%) |
| **LYCOPENE** | 2 (0.4%) | 3 (0.8%) | 7 (1.6%) | 1 (0.4%) | 6 (1.8%) | 4 (0.8%) | 23 (0.9%) |
| **METFORMIN** | 35 (7.2%) | 35 (9%) | 40 (9.4%) | 23 (8.1%) | 24 (7.2%) | 52 (9.8%) | 209 (8.5%) |
| **NITRATE** | 9 (1.8%) | 16 (4.1%) | 8 (1.9%) | 10 (3.5%) | 12 (3.6%) | 26 (4.9%) | 81 (3.3%) |
| **NON_LIPOPHILIC_STATIN** | 11 (2.3%) | 22 (5.7%) | 5 (1.2%) | 10 (3.5%) | 13 (3.9%) | 25 (4.7%) | 86 (3.5%) |
| **NSAID** | 112 (23%) | 152 (39.3%) | 97 (22.7%) | 109 (38.2%) | 140 (41.9%) | 214 (40.5%) | 824 (33.6%) |
| **PRAZOLE** | 136 (27.9%) | 171 (44.2%) | 144 (33.7%) | 115 (40.4%) | 137 (41%) | 219 (41.4%) | 922 (37.6%) |
| **SELENIUM** | 4 (0.8%) | 15 (3.9%) | 17 (4%) | 5 (1.8%) | 2 (0.6%) | 7 (1.3%) | 50 (2%) |
| **SITAGLIPTIN** | 5 (1%) | 1 (0.3%) | 3 (0.7%) | 1 (0.4%) | 0 (0%) | 0 (0%) | 10 (0.4%) |
| **STATIN** | 79 (16.2%) | 105 (27.1%) | 104 (24.4%) | 54 (18.9%) | 67 (20.1%) | 119 (22.5%) | 528 (21.6%) |
| **SULFONLUREA** | 17 (3.5%) | 24 (6.2%) | 21 (4.9%) | 20 (7%) | 11 (3.3%) | 59 (11.2%) | 152 (6.2%) |
| **TOCOPHEROL** | 0 (0%) | 10 (2.6%) | 0 (0%) | 8 (2.8%) | 3 (0.9%) | 6 (1.1%) | 27 (1.1%) |
| **VERAPAMIL** | 4 (0.8%) | 3 (0.8%) | 7 (1.6%) | 4 (1.4%) | 5 (1.5%) | 8 (1.5%) | 31 (1.3%) |
| **VIT_C** | 2 (0.4%) | 36 (9.3%) | 1 (0.2%) | 14 (4.9%) | 11 (3.3%) | 12 (2.3%) | 76 (3.1%) |
| **WARFARIN** | 26 (5.3%) | 45 (11.6%) | 70 (16.4%) | 9 (3.2%) | 32 (9.6%) | 34 (6.4%) | 216 (8.8%) |
