# Supplementary material for: Assessment of a prognostic model, PSA metrics and toxicities in metastatic castrate resistant prostate cancer using data from Project Data Sphere (PDS)
Source: PLoS One. 2017 Feb 2;12(2):e0170544. doi: 10.1371/journal.pone.0170544 (PMC5289419; doi:10.1371/journal.pone.0170544)
Supplement: S3 Table — Baseline clinical factors in the 6 trials from Project Data Sphere. (DOC) [file pone.0170544.s005.doc]

S3 Table.

|  |  | **Study** | | | | | | |
| --- | --- | --- | --- | --- | --- | --- | --- | --- |
| ***variable*** | ***Category*** | *Celgene_2009_90* | *CougarB_2008_101* | *Novacea_2006_89* | *Pfizer_2008_81* | *Sanofi_2007_79* | *Sanofi_2007_83* | *All* |
| **Age, years** | *Median (IQR)* | 68 (63-73) | 69 (63-75) | 71.3 (65.8-77.1) | 67 (62-72) | 67 (61-73) | 67 (62-73) | 68 (63-74) |
|  | *Missing* | 0 (0%) | 3 (0.8%) | 0 (0%) | 0 (0%) | 0 (0%) | 0 (0%) | 3 (0.1%) |
| **BSA,** 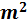 | *Median (IQR)* | 2 (1.9-2.1) | 2 (1.9-2.1) | 2 (1.9-2.1) | 1.9 (1.8-2.1) | 2 (1.9-2.1) | 2 (1.8-2.1) | 1.98 (1.863-2.11) |
|  | *Missing* | 7 (1.4%) | 0 (0%) | 2 (0.5%) | 4 (1.4%) | 34 (10.1%) | 1 (0.2%) | 48 (2%) |
| **Race** | *ASIAN* | 0 (0 %) | 9 (2.3 %) | - | 28 (9.8 %) | 23 (7.6 %) | 30 (5.7 %) | 90 (4.6%) |
|  | *BLACK* | 24 (5.5 %) | 15 (3.9 %) | - | 12 (4.2 %) | 18 (5.9 %) | 14 (2.6 %) | 83 (4.3%) |
|  | *OTHER* | 12 (2.7 %) | 5 (1.3 %) | - | 31 (10.9 %) | 11 (3.6 %) | 7 (1.3 %) | 66 (3.4%) |
|  | *WHITE* | 402 (91.8 %) | 358 (92.5 %) | - | 214 (75.1 %) | 251 (82.8 %) | 478 (90.4 %) | 1703 (87.7%) |
|  | *Missing* | 49 (10.1%) | 0 (0%) | 427 (100%) | 0 (0%) | 31 (9.3%) | 0 (0%) | 507 (20.7%) |
| **Metastasis location** | *Bone* | 309 (63.4 %) | 159 (41.1 %) | 296 (69.3 %) | 171 (60 %) | 187 (56 %) | 347 (65.6 %) | 1469 (60%) |
|  | *Liver* | 20 (4.1 %) | 29 (7.5 %) | 5 (1.2 %) | 29 (10.2 %) | 28 (8.4 %) | 51 (9.6 %) | 162 (6.6%) |
|  | *Lung* | 45 (9.2 %) | 35 (9 %) | 7 (1.6 %) | 40 (14 %) | 39 (11.7 %) | 56 (10.6 %) | 222 (9.1%) |
|  | *Lymph Node* | 41 (8.4 %) | 0 (0 %) | 97 (22.7 %) | 11 (3.9 %) | 19 (5.7 %) | 35 (6.6 %) | 203 (8.3%) |
|  | *Other* | 51 (10.5 %) | 158 (40.8 %) | 22 (5.2 %) | 31 (10.9 %) | 56 (16.8 %) | 40 (7.6 %) | 358 (14.6%) |
|  | *Missing* | 21 (4.3%) | 6 (1.6%) | 0 (0%) | 3 (1.1%) | 5 (1.5%) | 0 (0%) | 35 (1.4%) |
| **Gleason Score** | *Median (IQR)* | - | 8 (7-9) | 7 (7-9) | - | - | 7 (7-9) | 7 (7-9) |
|  | *Missing* | 487 (100%) | 45 (11.6%) | 30 (7%) | 285 (100%) | 334 (100%) | 20 (3.8%) | 1201 (49%) |
| **PSA, ng/Ml** | *Median (IQR)* | 75.4 (30-245.8) | 142.4 (41.1-433.7) | 63.3 (24-191.1) | 129 (38.8-375.2) | 100.4 (37.4-331) | 84.2 (29.9-225.6) | 88.7 (32.3-279.9) |
|  | *Missing* | 9 (1.8%) | 21 (5.4%) | 32 (7.5%) | 9 (3.2%) | 5 (1.5%) | 4 (0.8%) | 80 (3.3%) |
| **ALBUMIN, g/ld.** | *Median (IQR)* | 4.3 (4.1-4.5) | 4.1 (3.9-4.4) | - | 4.2 (3.9-4.4) | - | 4.1 (3.8-4.4) | 4.2 (3.9-4.412) |
|  | *Missing* | 6 (1.2%) | 2 (0.5%) | 427 (100%) | 7 (2.5%) | 334 (100%) | 21 (4%) | 797 (32.5%) |
| **HEMOGLOBIN, g/ld.** | *Median (IQR)* | 12.7 (11.7-13.7) | 11.9 (10.8-12.8) | 12.5 (11.6-13.5) | 12.1 (10.9-13.1) | 12.3 (11.3-13.2) | 12.8 (11.8-13.6) | 12.5 (11.4-13.4) |
|  | *Missing* | 23 (4.7%) | 21 (5.4%) | 33 (7.7%) | 11 (3.9%) | 8 (2.4%) | 6 (1.1%) | 102 (4.2%) |
| **LDH, U/L** | *Median (IQR)* | 206 (173.5-269.5) | 235.5 (187.8-326.2) | 202 (176-249) | 218 (182-302) | - | - | 214 (179-283) |
|  | *Missing* | 8 (1.6%) | 15 (3.9%) | 24 (5.6%) | 12 (4.2%) | 334 (100%) | 529 (100%) | 922 (37.6%) |
| **ALP, U/L** | *Median (IQR)* | 119 (78.8-244.5) | 133.5 (84-276.2) | 112.5 (78-213) | 124 (75-232) | 134 (85-252) | 132 (83-244) | 124 (81-243) |
|  | *Missing* | 7 (1.4%) | 3 (0.8%) | 9 (2.1%) | 7 (2.5%) | 11 (3.3%) | 8 (1.5%) | 45 (1.8%) |
| **Neutrophil, 10*3/UL** | *Median (IQR)* | 4.4 (3.4-5.7) | 4.3 (3.3-5.8) | 4.3 (3.3-5.6) | 4.4 (3.4-5.8) | 4.3 (3.3-5.6) | 4.2 (3.4-5.4) | 4.34 (3.34-5.66) |
|  | *Missing* | 31 (6.4%) | 20 (5.2%) | 33 (7.7%) | 14 (4.9%) | 6 (1.8%) | 8 (1.5%) | 112 (4.6%) |
| **Lymphocyte, 10*3/UL** | *Median (IQR)* | 1.4 (1.1-1.9) | 1.1 (0.8-1.5) | - | 1.3 (1-1.7) | 1.3 (1-1.8) | - | 1.28 (0.94-1.775) |
|  | *Missing* | 31 (6.4%) | 20 (5.2%) | 427 (100%) | 14 (4.9%) | 9 (2.7%) | 529 (100%) | 1030 (42.1%) |
| **Neutrophil-to-lymphocyte ratio** | *Median (IQR)* | 3.1 (2.2-4.3) | 4 (2.7-6.5) | - | 3.4 (2.4-5.1) | 3.2 (2.2-5.1) | - | 3.357 (2.326-5.166) |
|  | *Missing* | 31 (6.4%) | 20 (5.2%) | 427 (100%) | 14 (4.9%) | 9 (2.7%) | 529 (100%) | 1030 (42.1%) |
| **Platelets,10*3/UL** | *Median (IQR)* | 276 (228-343.5) | 249 (200-312) | 252 (206-306) | 238 (194-300) | 254.5 (203-305.2) | 247 (206-302) | 254 (206-312) |
|  | *Missing* | 28 (5.7%) | 21 (5.4%) | 42 (9.8%) | 16 (5.6%) | 33 (9.9%) | 8 (1.5%) | 148 (6%) |
| **Platelet-to-lymphocyte ratio** | *Median (IQR)* | 190.1 (138.5-275.7) | 219.4 (160.9-342) | - | 189 (133.9-278.2) | 194.8 (126.4-282.1) | - | 198.5 (139.1-296.4) |
|  | *Missing* | 36 (7.4%) | 22 (5.7%) | 427 (100%) | 20 (7%) | 41 (12.2%) | 529 (100%) | 1075 (43.9%) |
| **WBC** | *Median (IQR)* | 6.7 (5.4-8.1) | 6 (5.1-7.2) | 6.6 (5.3-7.9) | 6.5 (5.4-8) | 6.6 (5.3-8.1) | 6.6 (5.4-8) | 6.56 (5.318-7.98) |
|  | *Missing* | 23 (4.7%) | 302 (78%) | 33 (7.7%) | 9 (3.2%) | 31 (9.3%) | 6 (1.1%) | 404 (16.5%) |
| **Creatinine, mg/ld.** | *Median (IQR)* | 0.9 (0.8-1.1) | 0.9 (0.7-1) | 1 (0.8-1.2) | 0.9 (0.7-1) | 0.9 (0.8-1.1) | 0.9 (0.8-1.1) | 0.905 (0.8-1.1) |
|  | *Missing* | 6 (1.2%) | 2 (0.5%) | 10 (2.3%) | 5 (1.8%) | 31 (9.3%) | 8 (1.5%) | 62 (2.5%) |
| **Urea, mg/ld.** | *Median (IQR)* | 18 (15-21) | - | - | 17 (14-21) | 13.5 (13.5-13.5) | 17.2 (14.5-21) | 17.26 (14.29-21) |
|  | *Missing* | 6 (1.2%) | 387 (100%) | 427 (100%) | 64 (22.5%) | 333 (99.7%) | 120 (22.7%) | 1337 (54.6%) |
| **Length of Follow-up, years** | *Median (IQR)* | - | 1.0 (.8-1.2) | 1.0 (.8-1.3) | 1.3 (.9-1.6) | 1.4 (1.1-1.7) | 2.5 (2.1-3.1) | .9 (1.2-1.8) |
